# Polyphenol Extract from *Phellinus igniarius* Protects against Acrolein Toxicity *In Vitro* and Provides Protection in a Mouse Stroke Model

**DOI:** 10.1371/journal.pone.0122733

**Published:** 2015-03-26

**Authors:** Papawee Suabjakyong, Ryotaro Saiki, Leo J. L. D. Van Griensven, Kyohei Higashi, Kazuhiro Nishimura, Kazuei Igarashi, Toshihiko Toida

**Affiliations:** 1 Department of Clinical and Analytical Biochemistry, Graduate School of Pharmaceutical Sciences, Chiba University, Chiba-shi, Chiba, Japan; 2 Amine Pharma Research Institute, Innovation Plaza at Chiba University, Chiba-shi, Chiba, Japan; 3 Plant Research International, Wageningen University and Research Centre, Wageningen, The Netherlands; Massachusetts General Hospital/Harvard Medical School, UNITED STATES

## Abstract

The basidiomycetous mushroom *Phellinus igniarius* (L.) Quel. has been used as traditional medicine in various Asian countries for many years. Although many reports exist on its anti-oxidative and anti-inflammatory activities and therapeutic effects against various diseases, our current knowledge of its effect on stroke is very limited. Stroke is a neurodegenerative disorder in which oxidative stress is a key hallmark. Following the 2005 discovery by Igarashi’s group that acrolein produced from polyamines *in vivo* is a major cause of cell damage by oxidative stress, we now describe the effects of anti-oxidative extracts from *P*. *igniarius* on symptoms of experimentally induced stroke in mice. The toxicity of acrolein was compared with that of hydrogen peroxide in a mouse mammary carcinoma cell line (FM3A). We found that the complete inhibition of FM3A cell growth by 5 μM acrolein could be prevented by crude ethanol extract of *P*. *igniarius* at 0.5 μg/ml. Seven polyphenol compounds named 3,4-dihydroxybenzaldehyde, 4-(3,4-dihydroxyphenyl-3-buten-2one, inonoblin C, phelligridin D, inoscavin C, phelligridin C and interfungin B were identified from this ethanolic extract by LCMS and ^1^H NMR. Polyphenol-containing extracts of *P*. *igniarius* were then used to prevent acrolein toxicity in a mouse neuroblastoma (Neuro-2a) cell line. The results suggested that Neuro-2a cells were protected from acrolein toxicity at 2 and 5 μM by this polyphenol extract at 0.5 and 2 μg/ml, respectively. Furthermore, in mice with experimentally induced stroke, intraperitoneal treatment with *P*. *igniarius* polyphenol extract at 20 μg/kg caused a reduction of the infarction volume by 62.2% compared to untreated mice. These observations suggest that the polyphenol extract of *P*. *igniarius* could serve to prevent ischemic stroke.

## Introduction

Mushrooms have been appreciated for their flavors, economic value, ecological value, and medicinal properties for many years [1]. Many therapeutic effects have been reported for medicinal mushrooms such as anti-inflammatory [2], antitumor [3–5], anticancer and immunomodulatory effects [6,7]; stimulating macrophage activity and anti-hepatitis B virus activity [8]; and anti-oxidative activities [9–12]. *Phellinus igniarius* (L.) Quélis, a basidiomycetous fungus belonging to the Hymenochaetaceae, has traditionally been used as a folk medicine due to its high biological activity [13]. *Phellinus igniarius* is a rich source of secondary metabolites like triterpenoids and polyphenols [14].

Polyphenol containing extracts from *Phellinus igniarius* were found to be strongly anti-oxidant [15–18]. Little or no attention has ever been paid to their effects on stroke, which is a neurodegenerative disorder in whose pathogenesis reactive oxygen species (ROS) [19] such as superoxide anion radical (O_2_^•^), hydrogen peroxide (H_2_O_2_), and hydroxyl radical (•OH) play an important role. Oxidative stress might be pathogenic at the early stage in the disease, and it can exacerbate it during later stages.

Iodoacetic acid (IAA) is an alkylating agent that reacts with cysteine residues of proteins. It is an irreversible inhibitor of glyceraldehyde 3-phosphate dehydrogenase (GAPDH), which is an enzyme of the glycolytic pathway. It was reported that IAA-treated neuronal cells die following depletion of intracellular ATP, mitochondrial dysfunction, and production of reactive oxygen species (ROS) [20–23]. These observations are similar with those of *in vivo* ischemic stroke [20].

Acrolein is a common environmental pollutant that is associated with respiratory disease, aberrant platelet aggregation and increased thrombosis [24]. Kazuei Igarashi’s group suggested that the toxicity of acrolein (CH_2_ = CHCHO) is more severe than that of H_2_O_2_ and nearly equal to that of •OH [25]. Acrolein is spontaneously formed from spermidine and spermine by amine oxidase [26]. Once cells are damaged, polyamines are released from RNA [27], and acrolein is produced by polyamine oxidizing enzymes. Acrolein is toxic and will cause further cell damage and increase the levels of protein-conjugated acrolein (PC-Acro) as observed in stroke [28]. Furthermore, Igarishi’s group presented that the toxicity of H_2_O_2_ was reduced by polyphenols [29]. Based on this we have investigated the effect of polyphenol extract from *Phellinus igniarius*, and it prevented toxicity from acrolein and ROS in FM3A cells, Neuro-2a cells and photo-induced thrombosis model mice. Furthermore, we identified *P*. *igniarius* polyphenols by liquid chromatography mass spectrometry (LCMS) and proton nuclear magnetic resonance. spectroscopy (NMR).

## Materials and Methods

### Mushroom extract preparation

Dried fruiting body powders of wild-type *Phellinus linteus* and *Phellinus igniarius* were kindly provided by Amazing Grace Health Products Limited Partnership (Bangkok, Thailand). They were extracted with 70% ethanol (10% w/v) at 70°C for 16 hrs. The supernatant was removed by centrifugation at 10,000 g for 20 min. Crude *Phellinus linteus* (PL EtOH) and *Phellinus igniarius* (PI EtOH) ethanol extracts were filtered and stored at -20°C for further use. Crude aqueous extracts were prepared by hot water extraction [30] for 16 hrs. The *Phellinus linteus* (PL DDW) and *Phellinus igniarius* (PI DDW) aqueous extracts were then filtered and stored at -20°C for further use. The *Phellinus linteus* (PL EtOH) and *Phellinus igniarius* (PI EtOH) ethanol extracts were lyophilized and stored at -20°C for further use. PI EtOH (PIO) and PL EtOH (PLO) powders were dissolved in water, filtered with a hydrophilic 0.22 μm filter, and stored at -20°C for MTT assays. PI EtOH (PIDMSO) and PL EtOH (PLDMSO) powders were dissolved in DMSO, filtered with a hydrophilic 0.22 μm filter, and stored at -20°C for MTT assays, mouse stroke model and analysis.

After ethanol extraction the tissue was further extracted with hot water [30] for 16 hrs. Crude polysaccharides were precipitated by addition of 2.5 volumes of cold 99% ethanol, after which the suspension was kept at -20°C for 16 to 24 hrs. The suspension was then centrifuged at 15,000 g for 20 minutes. The precipitate was dissolved in a small volume of water and the polysaccharides were reprecipitated with alcohol as before for 4–5 times. Ethanol-soluble phenolic compounds were removed by a Sep-Pak C18 Plus Light Cartridge. The polysaccharides of *Phellinus linteus* (PLP) and *Phellinus igniarius* (PIP) were lyophilized and stored at -20°C for further use.

### Analysis of polyphenols by liquid chromatography mass spectrometry (LCMS)

Chromatographic separation was performed on a Hitachi LaChrom Elite HPLC ultra-high-pressure liquid chromatography (U-HPLC) system (Hitachi High-Technologies Corporation, Tokyo, Japan) fitted with a Bruker amaZon SL Ion Trap Mass Spectrometer. The Chromatographic separation was performed on an Inertsil ODS-3 150 mm × 4.6 mm, 5 μm (GL Sciences Inc., Tokyo, Japan). The mobile phase was composed of water (A) with formic acid solution (pH = 3.0) and 100% acetonitrile (B) with formic acid solution (pH = 3.0) under gradient elution conditions at 0–60 min, 0–50% B; 60–90 min, 50–100% B and 90–100 min, 100% B. The flow rate of the mobile phase was 0.4 ml/min, and the column temperature was maintained at 30°C. Mass spectrum analysis was performed using a Bruker amaZon SL Ion Trap Mass Spectrometer (Bruker, Yokohama, Japan) fitted with an ESI source and operated in negative ion mode. The key optimized ESI parameters were as follows: spray voltage: -3.5 KV; capillary temp: 220°C; Ultra scan. Data were collected with Bruker Compass Data Analysis 4.2 (Bruker, Yokohama, Japan) and analyzed by Mass++ software [31] with the MassBank mass spectral library.

### Nuclear magnetic resonance spectroscopy (NMR)

One-dimensional spectra on 10 mg of dry sample exchanged in dimethyl sulfoxide-d6, 99.9% containing 0.05 v/v TMS (Wako, Japan) were acquired using a Jeol 600 MHz instrument. The operation conditions for ^1^H NMR were as follows: spin, 15 Hz; relaxation delay, 5 s; temperature, 25°C.

### Cell culture

Mouse mammary carcinoma FM3A cells [32] (5×10^4^ cells/ml) were cultured in low glucose DMEM (Dulbecco’s modified Eagle’s medium; Nacalai Tesque, Inc., Japan) under 6 conditions: control (standard conditions); samples alone at 50, 5 and 0.5 μg/ml; samples with acrolein at 10 μM, 5 and 2 μM; only acrolein; samples with H_2_O_2_ at 200, 150 and 100 μM; and only H_2_O_2_ for 24 and 48 hrs.

Mouse neuroblastoma cells (Neuro-2a cells) [33] were grown in DMEM culture medium (Nacalai Tesque, Inc., Japan) supplemented with 10% fetal bovine serum (Gibco, USA) and 1% penicillin-streptomycin mixed solution (Nacalai Tesque, Inc., Japan) at 37°C in 5% CO_2_ in a humidified incubator. Neuro-2a cells (3×10^4^ cells/ml) were plated in 96-well microtiter plates in DMEM medium under the standard conditions for 12 hrs. Then, the Neuro-2a cells were treated with samples and medium for a control. The activity of the Neuro-2a cells was then measured by MTT assay.

### Trypan blue assay

Viability of cells was tested in culture fluid with 0.25% trypan blue. After 1 minute the cells were counted under a microscope and unstained cells were considered alive. Cell counts were done in triplicate.

### MTT assay

Cells were plated in 96-well microtiter plates and treated as described above. Thereafter, the medium was removed and 50 μl/ml of MTT solution in DMEM and antibiotics (penicillin and streptomycin) were added. After a 1 hr incubation, the MTT solution was replaced with 100 μl of DMSO to dissolve the tetrazolium crystals. Finally, the absorption was read at a test wavelength of 540 nm and a reference wavelength of 650 nm with a Multiskan JX microplate reader (Thermo Labsystems, UK). Cell viability (%) was calculated as [optical density (OD) of the treated wells]/[OD of the control wells] x 100 [34].

### Photochemically Induced Thrombosis Model Mice

All animal experiments were approved by the Institutional Animal Care and Use Committee of Chiba University and carried out according to the Guidelines for Animal Research of Chiba University. Male C57BL/6NCrSlc mice (8-week-old) were purchased from Japan SLC Inc. (Japan). Nine-week-old mice weighing 22 to 27 g were anesthetized with inhalation of 3% isoflurane (Abbott, Japan). Anesthesia was continued with 2% isoflurane during the operation. A thrombotic occlusion of the middle cerebral artery was induced by a photochemical reaction [35]: an incision was made between the left orbit and the external auditory canal, and the temporalis muscle was detached from the dura mater to expose the proximal section of the middle cerebral artery. Immediately after intravenous injection of a photosensitizer, Rose Bengal (20 mg/kg), through a jugular vein, green light (wavelength: 540 nm) emitted from a xenon lamp (Hamamatsu Photonics, Japan) illuminated the middle cerebral artery for 10 min. After middle cerebral artery occlusion, the incised skin was restored. At 24 hrs after the induction of photochemically induced thrombosis (PIT) stroke, we took blood from the anesthetized mice via the cardiac puncture method. After that, the brain was removed and sectioned into 2-mm thick coronal slices. Each slice was incubated with 5% triphenyltetrazolium chloride solution at 37°C for 10 min. The volume of the infarction was analyzed using ImageJ software [36]. Where indicated, *P*. *igniarius* ethanol extract dissolved in DMSO (PIDMSO) at 200 ng to 20 mg/kg in phosphate-buffered saline was injected intraperitoneally at 0 hr after infarction induction. The experiments were performed using 6 mice in each group [37].

### Measurement of PC-Acro

Brain tissues at the locus of infarction in the PIT model mice and at the same locus in control mice were homogenized using an Ultra-Turrax homogenizer in 0.5 ml of buffer A containing 10 mmol/L Tris/HCl (pH 7.5), 1 mmol/L dithiothreitol, 10% glycerol, 0.2 mmol/L EDTA, and 0.02 mmol/L FUT-175 (6-amino-2-naphthyl-4-guanidinobenzoate), a protease inhibitor [38]. Total proteins (5 μg) were stained with Coomassie Brilliant Blue R-250 after sodium dodecyl sulfate–polyacrylamide gel electrophoresis, and the level of PC-Acro was measured by Western blotting [39] using 5 μg of tissue homogenate protein and a polyclonal antibody against bovine serum albumin-conjugated acrolein (MoBiTec, Germany). The level of albumin was measured similarly using an antibody against bovine serum albumin-conjugated acrolein mouse albumin (Bethyl, USA). Relative levels of PC-Acro and albumin were estimated by measuring the density of bands of 6 mice with a LAS-1000 imaging analyzer (Fuji film, Japan). Cardiac blood containing 3 U/ml heparin was centrifuged at 3,000 g for 10 minutes at 4°C. The supernatant (plasma) was carefully collected to avoid contamination by erythrocytes. PC-Acro, in which acrolein is converted to N^Ɛ^-(3-formyl-3,4-dehydropiperidino-lysine) (FDP-lys) in plasma, was measured by the ACR-LYSINE ADDUCT ELISA system (NOF Corporation) using 0.05 ml of plasma [40]. After the reaction was terminated, absorbance at 450 nm was measured by a Bio-Rad Model 550 microplate reader. Protein concentrations were determined by the method of Bradford et al [41].

### Statistics

Statistical analyses were carried out using GraphPad Prism version 4.0 for Windows 7 (GraphPad,USA). ANOVA with Tukey's multiple comparison test was applied for multiple comparisons. Values are indicated as means ± SE, and significant differences are shown as probability values.

## Results

### Acrolein toxicity *in vitro*

To characterize the effects of PI EtOH extract on acrolein treated FM3A cells we first determined the possible cytotoxicity of PI EtOH extract by trypan blue assay. The extract showed no cytotoxicity at concentrations of 2, 1 and 0.5 μg/ml and only very slight cytotoxicity at 5 μg/ml.

**Fig 1:**
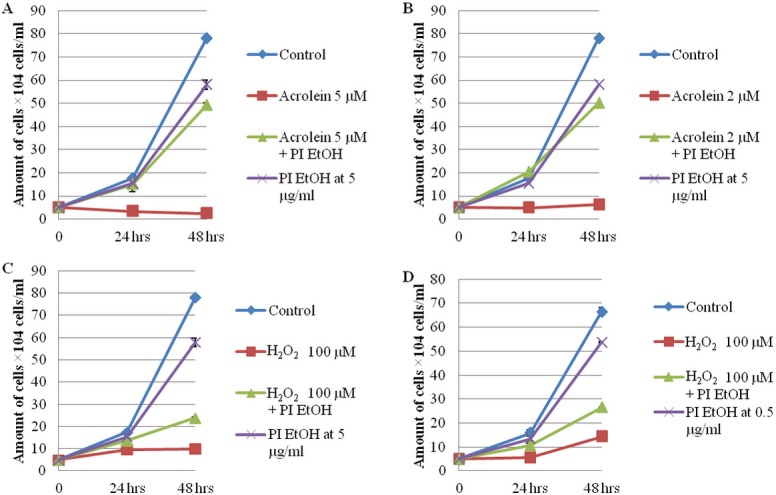
The cytotoxicity of acrolein and H_2_O_2_ in FM3A cells. The viable cell number of FM3A cells that were treated with PI EtOH extract at 5 μg/ml plus acrolein at 5 μM (A) or 2 μM (B) or 100 μM H_2_O_2_ and cultured for 24 and 48 hrs. The number of cells was counted with 0.25% trypan blue. Each value represents the mean ± SD (n = 3).

The cell toxicity caused by 5 (Fig 1A) and2 μM (Fig 1B) acrolein was strongly prevented by 5 μg/ml PI EtOH extract and also prevented by a lower concentration of PI EtOH extract (data not shown). Cell toxicity caused by 100 μM H_2_O_2_ was prevented by 5 μg/ml PI EtOH extract (Fig 1C), and it was also prevented by 0.5 μg/ml PI EtOH extract (Fig 1D). The results further showed that PI EtOH extract could not degrade higher concentrations of H_2_O_2_ (data not shown).

**Fig 2:**
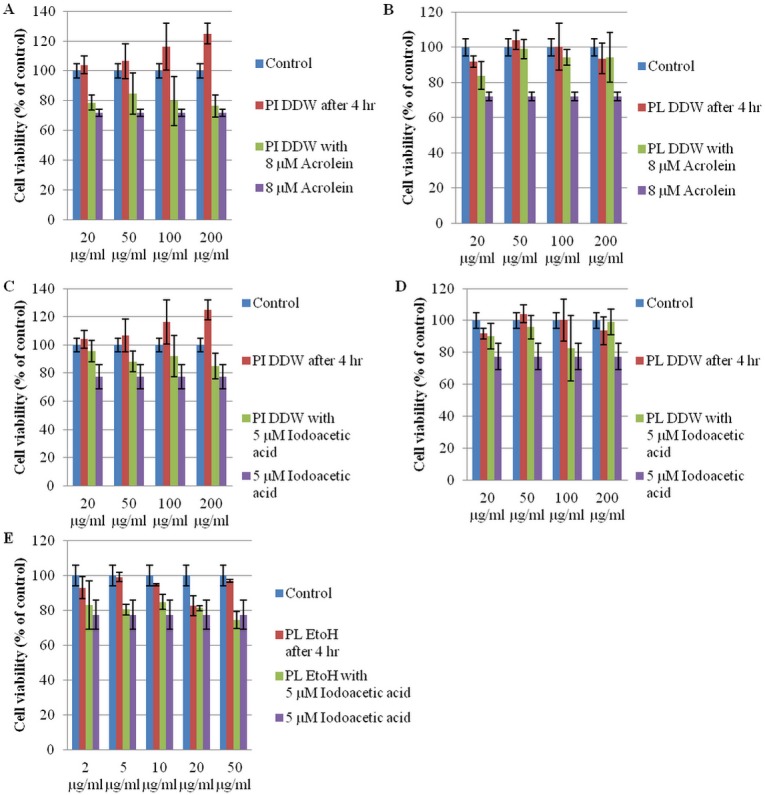
The effect of *P*. *igniarius* and *P*. *linteus* extracts on the cytotoxicity of acrolein and iodoacetic acid. The viable cell number of Neuro-2a cells that were treated with PI (A) or PL (B) DDW crude extract at 20, 50, 100 and 200 μg/ml after a pretreatment with 8 μM acrolein for 4 hrs. PI (C) and PL (D) DDW crude extract at 20, 50, 100 and 200 μg/ml after a pretreatment with 5 μM iodoacetic acid for 4 hrs. PL EtOH crude extract (E) at 2, 5, 10, 20 and 50 μg/ml after a pretreatment with 5 μM iodoacetic acid for 4 hrs. Cell viability was determined by MTT assay after 24 hrs. Each value represents the mean ± SD (n = 3).

**Fig 3:**
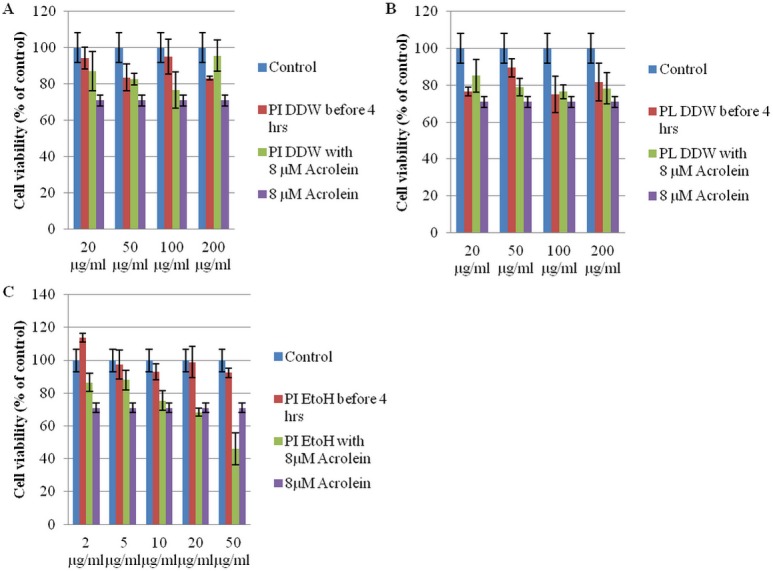
Effect of pretreatment of Neuro-2a cells with PL and PI extracts on cytotoxicity of acrolein. The viable cell number of Neuro-2a cells that were treated with PI EtOH crude extract (A) at 2, 5, 10, 20 and 50 μg/ml, PI (B) or PL (C) DDW crude extract at 20, 50, 100 and 200 μg/ml for 4 hrs and treated with 8 μM acrolein. Cell viability was determined by MTT assay after 24 hrs. Each value represents the mean ± SD (n = 3).

We therefore undertook to measure the cytotoxicity of acrolein and iodoacetic acid in Neuro-2a cells instead of the solid tumor derived FM3A cells. The results showed that PL and PI crude aqueous extracts may protect cultured Neuro-2a cells, i.e., the number of cells at the end of the incubation period, from the deleterious effects of being pretreated with 8 μM acrolein or 5 μM iodoacetic acid, respectively, for 4 hrs. PL ethanol extract may allow cells to recover after a pretreatment with 5 μM iodoacetic acid for 4 hrs (Fig 2). In Fig 3, it is shown that crude polysaccharide extract from *Phellinus linteus* and *Phellinus igniarius* provided no protection against iodoacetic acid. On the other hand, 0.5 μM PI EtOH extract and PI- and PL-derived polysaccharide extracts can protect cells from the toxic effects of 8 μM acrolein for 24 hrs (Fig 3). These results strongly suggest that PI- and PL-derived extracts can protect Neuro-2a cells from acrolein cytotoxicity.

**Fig 4:**
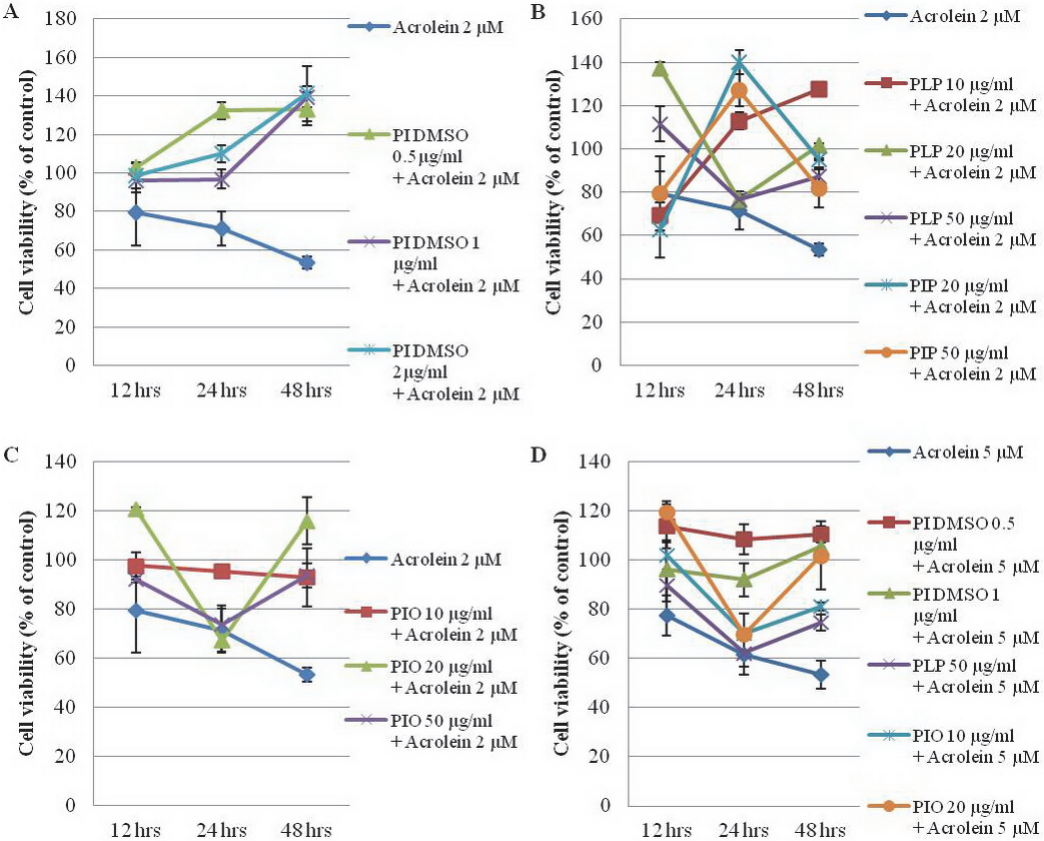
Matrix assay of ethanol extract. EtOH extract dissolved in DMSO at 0.5, 1 and 2 μg/ml with acrolein at 2 μM (A); glucans of PI at 20 and 50 μg/ml and polysaccharides of PL at 10, 20 and 50 μg/ml with acrolein at 2 μM (B); PI ethanol extract dissolved in DDW at 10, 20 and 50 μg/ml with acrolein at 2 μM (C) or 5 μM (D). Cell viability was determined by MTT assay after 12, 24 and 48 hrs. Each value represents the mean ± SD (n = 3).

We then did a time study, which is shown in Figs 3 and 4. Crude polysaccharides of *Phellinus linteus* and *Phellinus igniarius* at 10, 20, 50 and 100 μg/ml, PI ethanol extract dissolved in water at 10, 20, 50 and 100 μg/ml and PI ethanol extract dissolved in DMSO at 0.5, 1, 2 and 5 μg/ml were tested on Neuro-2a cells with acrolein at 2, 5, and 10 μM for 12, 24 and 48 hrs.

The results showed that PI ethanol extract dissolved in DMSO at 0.5, 1 and 2 μg/ml protected Neuro-2a cells from the effects of 2 μM acrolein. Also, PL polysaccharides at 10, 20 and 50 μg/ml and PI ethanol extract dissolved in water at 10, 20 and 50 μg/ml may protect cells from 2 μM acrolein. PI ethanol extract dissolved in DMSO at 0.5 and 1 μg/ml may protect cells from 5 μM acrolein. PL polysaccharides at 50 μg/ml may protect cells from 5 μM acrolein (Fig 4).

### Protection in a mouse stroke model

**Fig 5:**
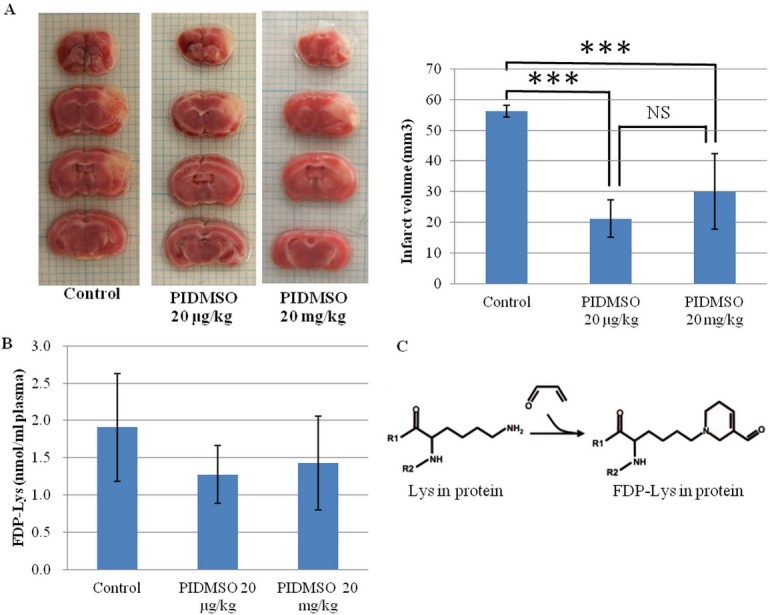
The effect of PIDMSO on a mouse stroke model. The effect of PIDMSO on brain infarction size (A), the level of PC-Acro in plasma (B) and the formation of FDP-lys from acrolein in protein (C). Experiments were performed using 6 mice in each group as described in Materials and Methods. Data are shown as mean ± SE. ***P<0.*001* compared with control mice.

**Fig 6:**
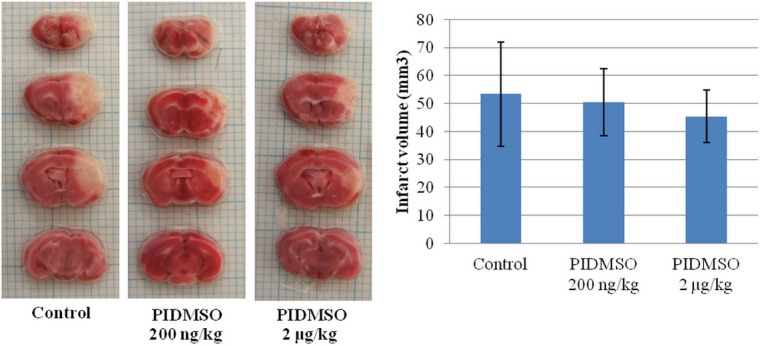
The effect of 200 ng/kg and 2 μg/kg PIDMSO on brain infarction size.

In the foregoing *in vitro* experiments we found that PIDMSO (= *P*. *igniarius* ethanol extract dissolved in DMSO) greatly reduced the inhibition of cell growth induced by acrolein. We therefore undertook to measure the effects *in vivo* using mice with brain infarction photochemically induced by Rose Bengal as a model The effects of PIDMSO on brain infarction were examined to confirm the correlation between brain infarction and PC-Acro (= protein-conjugated acrolein). Mice were injected intraperitoneally with PIDMSO at 200 ng to 20 mg/kg. A brain infarction was photo-induced after injection of Rose Bengal [35]. The volume of the infarction was determined by staining 2 mm thick coronal slices with triphenyltetrazolium. This stains the viable brain tissue red, whereas infarct tissue remains unstained [42]. Under our experimental conditions, in which we measured the average volume of infarction at 24 hrs after induction of stroke, the average volume of infarction decreased from 56.22 mm^3^ to 21.33 mm^3^ and 30.02 mm^3^ from intraperitoneally applied PIDMSO at 20 μg/kg and 20 mg/kg, respectively (Fig 5A). The stroke-induced mice treated intraperitoneally with 20 μg/kg PIDMSO showed the highest reduction of infarction volume, 62.24%. Furthermore, stroke-induced mice treated intraperitoneally with 200 ng/kg and 2 μg/kg PIDMSO experienced infarction volume reductions of 5.33% and 14.94%, less than the infarction volume reductions with 20 μg/kg to 20 mg/kg PIDMSO (Fig 6). However, the infarction volume reduction did not change significantly with 20 μg/kg, 200 μg/kg, 2 mg/kg and 20 mg/kg PIDMSO (data not shown).

**Fig 7:**
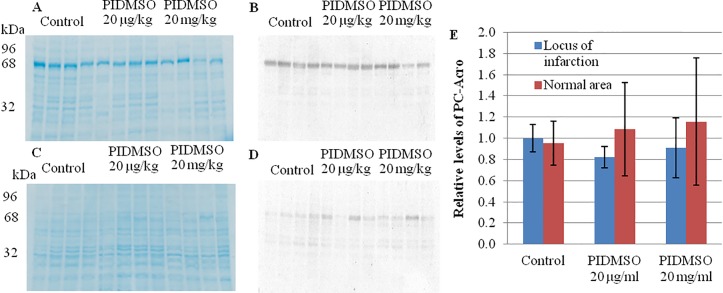
The levels of PC-Acro and albumin in brain tissue. Total proteins (5 μg) from brain tissues at the locus of infarction (A,B) and a normal area (C,D) were stained with Coomassie Brilliant Blue R250 (A,C), and the relative levels of PC-Acro and albumin were estimated by Western blotting using antibodies against mouse albumin (B,D,E). The results represent the mean ± SD of 6 mice.

Free acrolein is rapidly converted to PC-Acro through its interaction with lysine side chains in proteins (Fig 5C) [43]. PC-Acro in plasma was measured by the ACR-LYSINE ADDUCT ELISA system. The level of PC-Acro in plasma decreased greatly (Fig 5B) concomitantly with a reduction of the infarction. Furthermore, PC-Acro at the locus of brain infarction was measured by Western blotting using an antibody against FDP-Lys. PC-Acro (68 kDa) at the locus of the brain infarction decreased modestly from intraperitoneal PIDMSO at 20 μg/kg and 20 mg/kg (Fig 7E).

### Characterization of polyphenol extract

**Fig 8:**
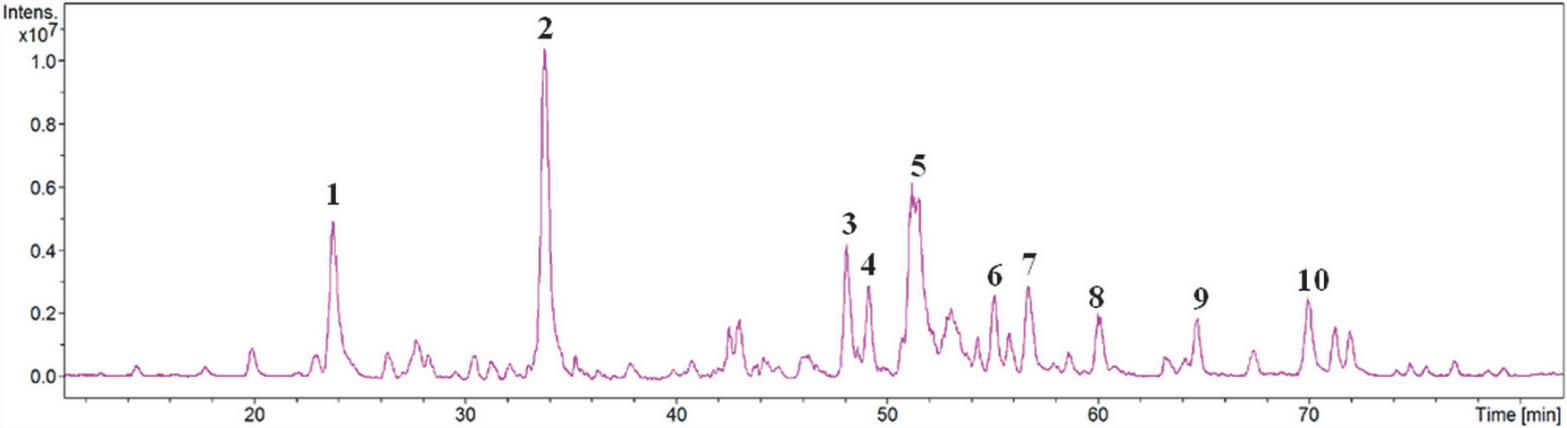
Total ion current (TIC) chromatogram of polyphenols from *Phellinus igniarius*.

To find out which compounds are prevalent in the PI ethanol extract we performed LCMS and NMR as described in Materials and Methods. The total ion current (TIC) chromatogram of the *Phellinus igniarius* ethanol extract showed 10 main peaks (Fig 8). The composition of the chromatograms was analyzed by MASS++ with the MassBank library. To confirm the identifications, the compounds were identified by comparing their retention time and mass spectral data (S1 Fig). The results suggest that the polyphenol extract from *Phellinus igniarius* mainly contains 3,4-dihydroxybenzaldehyde, 4-(3,4-dihydroxyphenyl-3-buten-2one, Inonoblin C, Phelligridin D, Inoscavin C, Phelligridin C and Interfungin B (Table 1). Furthermore, ^1^H NMR resonances of polyphenols were assigned, identifying Inonoblin C [44] (compounds 3 and 4), Phelligridin D [44] (compound 5), Inoscavin C [45] (compound 6) and Phelligridin C [45] (compound 7) in Table 2.

**Table 1:** Tentative identification of polyphenol extract from *Phellinus igniarius* by UPLC/ESI-MS in negative.

Compounds	RT (min)	Tentative identification	MW	[M-H]^-^ m/z	UPLC/ESI-MS (% base peak)
1	23.8	3,4-dihydroxybenzaldehyde	138.12	136.81	136.81(100), 274.86(14.27), 342.94(5.98)
2	33.8	4-(3,4-dihydroxyphenyl-3-buten-2one	178.19	176.81	176.81(100), 355.01(16.71)
3	48.0	inonoblin C	462.42	461.07	461.07(100), 196.79(6.10)
4	49.1	inonoblin C	462.42	461.07	461.07(100), 196.79(6.10)
5	51.2	phelligridin D	380.31	378.98	378.98(100), 492.97(6.69)
6	55.2	inoscavin C	420.38	419.06	419.06(100), 310.98(11.18), 758.17(2.60), 454.98(6.93), 847.25(6.49), 453.07(6.48), 375.01(6.34), 411.10(5.83), 459.04(5.77), 381.07(5)
7	56.7	phelligridin C	364.31	363.01	363.01(100), 379.00(5.57)
8	60	unknown	n.d.	262.95	262.95(100), 571.02(15.03), 218.93(14.74), 360.98(13.51), 321.02(12.51), 419.05(9.56), 385.95(8.05), 359.11(7.97), 343.06(7.10),379.00(6.56), 250.91(6.46), 364.97(5.78), 751.02(5.76)
9	64.7	interfungin B	422.40	421.12	421.12(100), 431.14(66.72), 459.08(9.10), 248.92(8.02), 265.03(6.06), 475.23(5.78), 771.24(5.77)
10	69.9	unknown	n.d.	246.90	246.90(100), 315.02(15.50), 871.16(9.39), 305.09(7.68), 433.05(5.47), 359.18(5.39), 309.01(5.07)

**Table 2:** Compounds identified in polyphenol extract from *Phellinus igniarius* by assignment of their proton resonances.

Compound	^1^H NMR chemical shifts and their assignments
3,4-Dihydroxybenzaldehyde **(1)**	9.702 s, 6.923 s, 6.909 s, 7.284 d (2.06), 7.270 d (2.06), 7.242 d (2.06)
Inonoblin C **(3),(4)**	6.161 s, 6.690 d (17.18), 7.330 d (17.18), 6.985 d (2.06), 6.751 d (8.25), 7.009 dd (8.25, 2.06), 6.816 s, 7.091 s, 6.260 brd (2.06), 2.280 s (Me)
Phelligridin D **(5)**	6.730 s, 7.539 s, 8,361 s, 6.690 d (17.18), 7.330 d (17.18), 7.068 d (2.06), 6.789 d (8.25), 7.009 dd (8.25, 2.06)
Inoscavin C **(6)**	6.816 s, 6.758 s, 7.091 s, 7.242 d (2.06), 6.847 d (8.25)
Phelligridin C **(7)**	6.730 s, 7.539 s, 8,361 s, 6.909 s, 6.847d (8.25)

## Discussion

In *in vitro* experiments, we observed that 5 μg/ml and lower concentrations of PI EtOH extract prevented the effects of 2 and 5 μM acrolein and also could protect against100 μM H_2_O_2_. PI EtOH extract strongly prevented the effects acrolein and to a higher extent than the effects of H_2_O_2_. The results suggest that the reactivity between •OH and PI EtOH extract may be relatively weak compared with the reactivity between acrolein and PI EtOH extract where strong prevention was observed. Furthermore, we observed that *Phellinus linteus* and *Phellinus igniarius* extracts can prevent acrolein cytotoxicity in Neuro-2a cells. However, *Phellinus linteus* and *Phellinus igniarius* extracts provided no protection against iodoacetic acid, which reacts with cysteine. Neuro-2a cells exposed to acrolein were treated with semi-purified extracts from *Phellinus linteus* and *Phellinus igniarius* and assessed. These observations indicated that the polyphenol extract of *P*. *igniarius* greatly reduced the growth inhibition of Neuro-2a cells that was caused by acrolein, contrary to earlier results by Kazuei Igarishi's group in which acrolein toxicity was not prevented by polyphenols [29]. The effects of polyphenols from *Phellinus igniarius* on artificially induced brain infarction showed that the average volume of infarction was significantly lower. Although the reduction of PC-Acro levels at the locus of the brain infarction and in plasma was not significant, the level of PC-Acro at the locus of brain infarction and in plasma had decreased and seemed related to the reduction of infarction volume.

**Fig 9:**
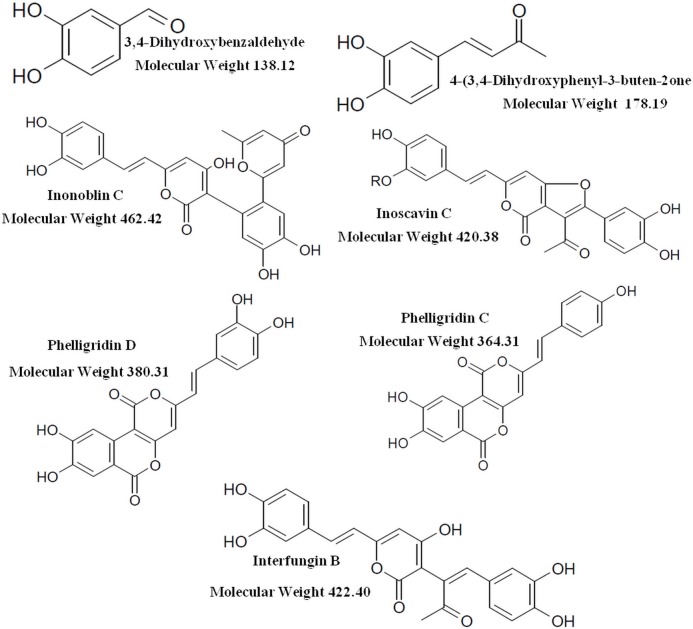
Structure of precursor polyphenols.

The polyphenol extract of *Phellinus igniarius* was isolated by DMSO extraction and analyzed by LCMS. The ESI-MS of compound 1 showed a 136.89 [M−H]^−^ m/z peak at 24.0 min (S1A Fig) whereas 3,4-dihydroxybenzaldehyde gave a 136.90 [M−H]^−^ m/z peak at 23.9 min (S2 Fig). The molecular formula of compound 1 was determined to be C_7_H_6_O_3_ by ^1^H NMR spectral data of 3,4-dihydroxybenzaldehyde (S4 Fig). This strongly suggested that compound 1 is 3,4-dihydroxybenzaldehyde (Table 1). The ESI-MS of compound 2 gave a 176.81 [M−H]^−^ m/z peak at 33.8 min (S1B Fig). The ESI-MS of esculetin and 4-(3,4-dihydroxyphenyl-3-buten-2one gave 176.75 (S3 Fig) and 177 [M−H]^−^ [46] m/z peaks, respectively, at 23.9 min. 4-(3,4-Dihydroxyphenyl-3-buten-2one has one more carbon atom than esculetin. It was tentatively suggested that compound 2 is 4-(3,4-dihydroxyphenyl-3-buten-2one. Compounds 3 to 7 and 9 were tentative identified by masses of the corresponding [M−H]^−^ from the M.Sc thesis of Weesepoel [46]. To confirm the identifications, the compounds were assigned proton resonances in polyphenols that present inonoblin C, phelligridin D, inoscavin C and phelligridin C by NMR. The polyphenol extract of *Phellinus igniarius* mainly contained 3,4-dihydroxybenzaldehyde, 4-(3,4-dihydroxyphenyl-3-buten-2one, inonoblin C, phelligridin D, inoscavin C, phelligridin C and interfungin B (Fig 9). Our observations suggest that the polyphenols of *Phellinus igniarius* could serve as a substance for a novel neuroprotective treatment of artificially induced ischemic stroke by scavenging the causal acrolein.

## Supporting Information

S1 Fig ESI-MS spectra from compounds 1–10 (A-J). (TIF)

S2 Fig Chromatogram and ESI-MS spectra of 3,4-dihydroxybenzaldehyde. (TIF)

S3 Fig Chromatogram and ESI-MS spectra of esculetin. (TIF)

S4 Fig ^1^H 600 NMR spectra of 3,4-dihydroxybenzaldehyde in DMSO-d6 with TMS at 25°C. (TIF)
