# Supplementary figures and images for: Polyphenol Extract from Phellinus igniarius Protects against Acrolein Toxicity In Vitro and Provides Protection in a Mouse Stroke Model
Source: PLoS One. 2015 Mar 26;10(3):e0122733. doi: 10.1371/journal.pone.0122733 (PMC4374876; doi:10.1371/journal.pone.0122733)

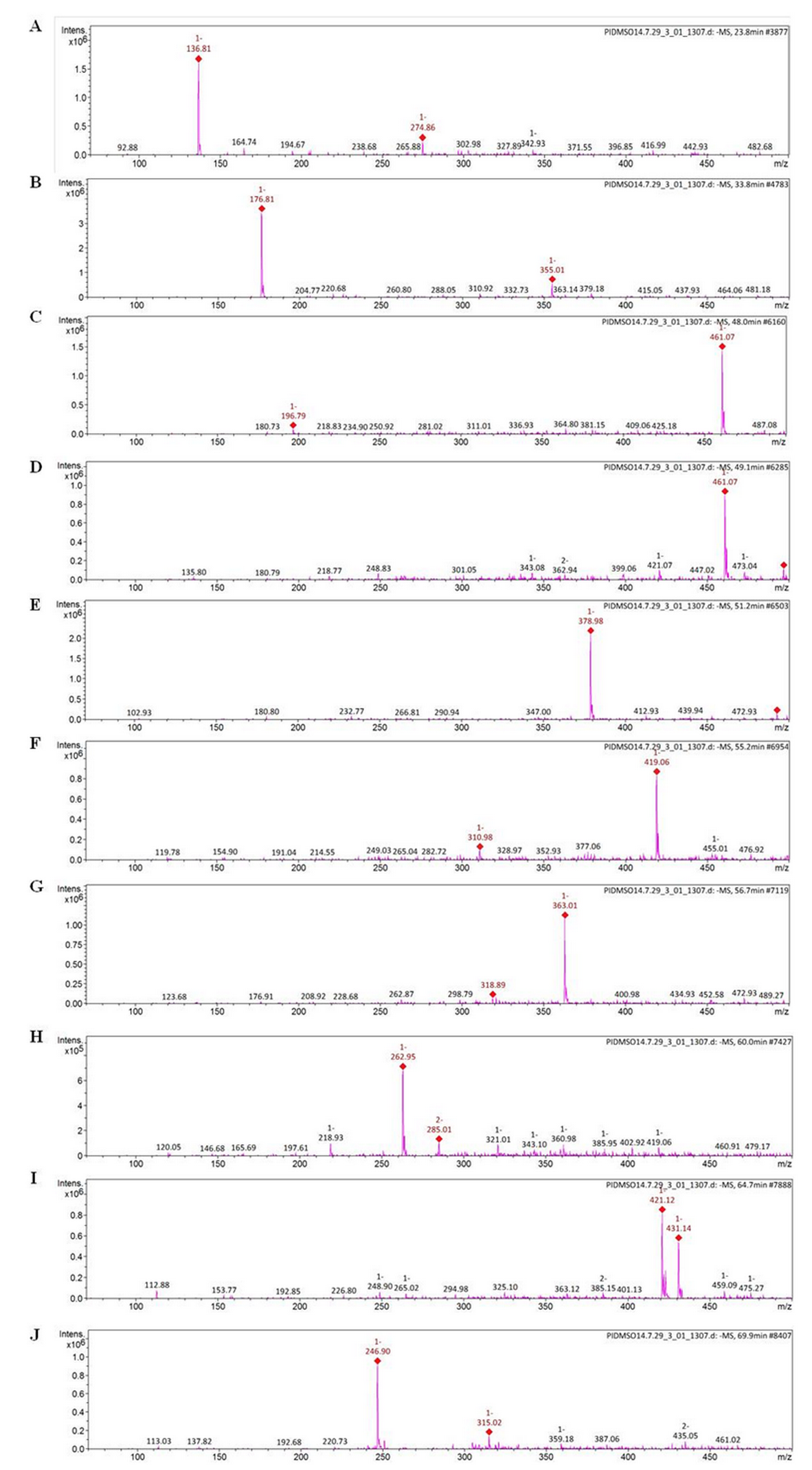

Supplement: S1 Fig — (TIF) [file pone.0122733.s001.tif]

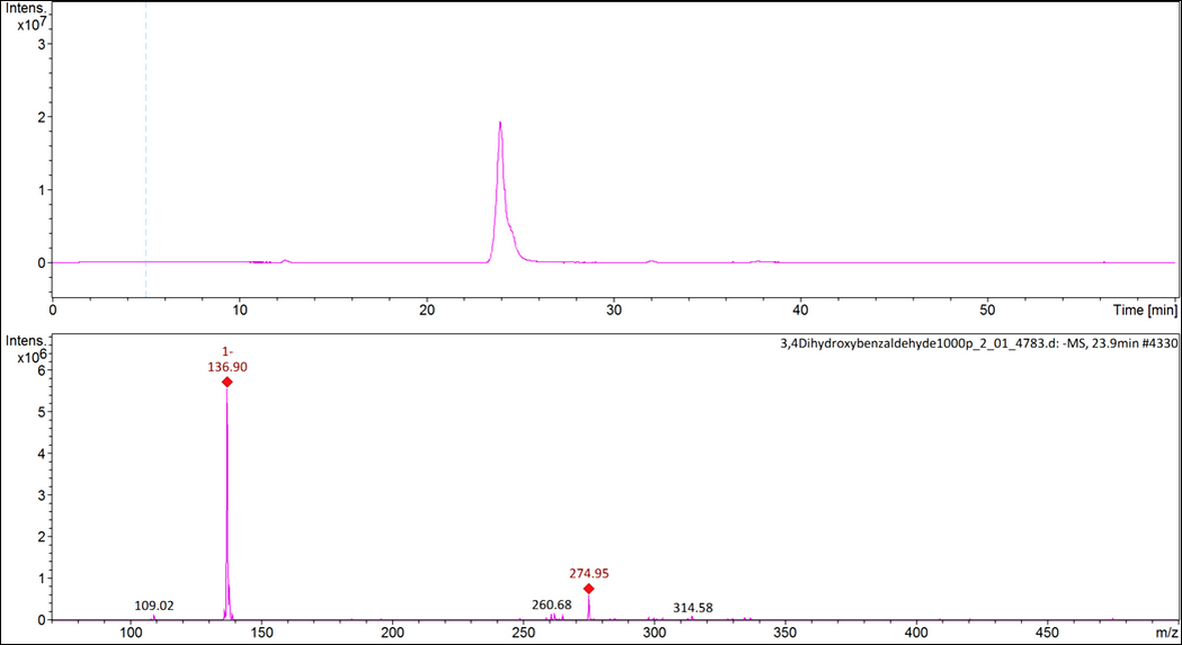

Supplement: S2 Fig — (TIF) [file pone.0122733.s002.tif]

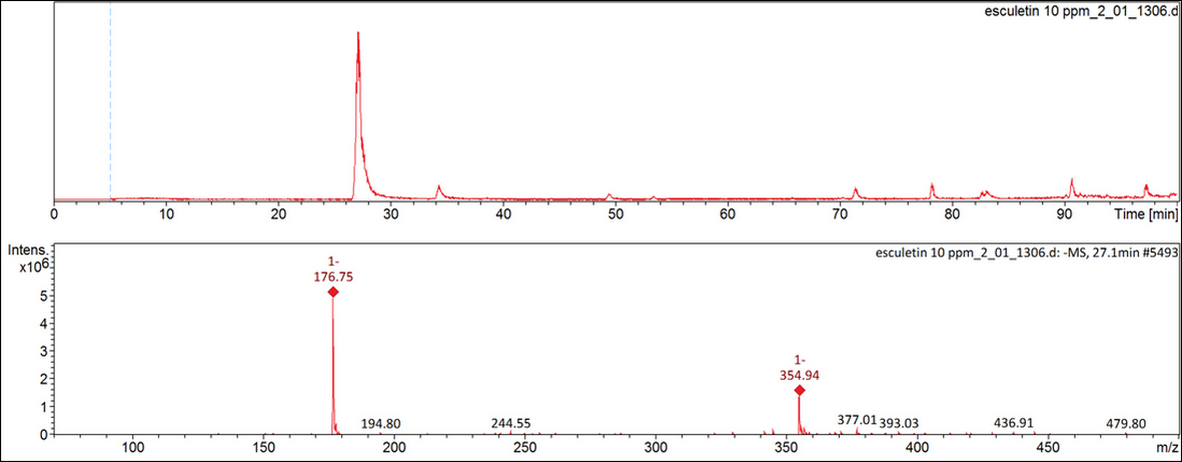

Supplement: S3 Fig — (TIF) [file pone.0122733.s003.tif]

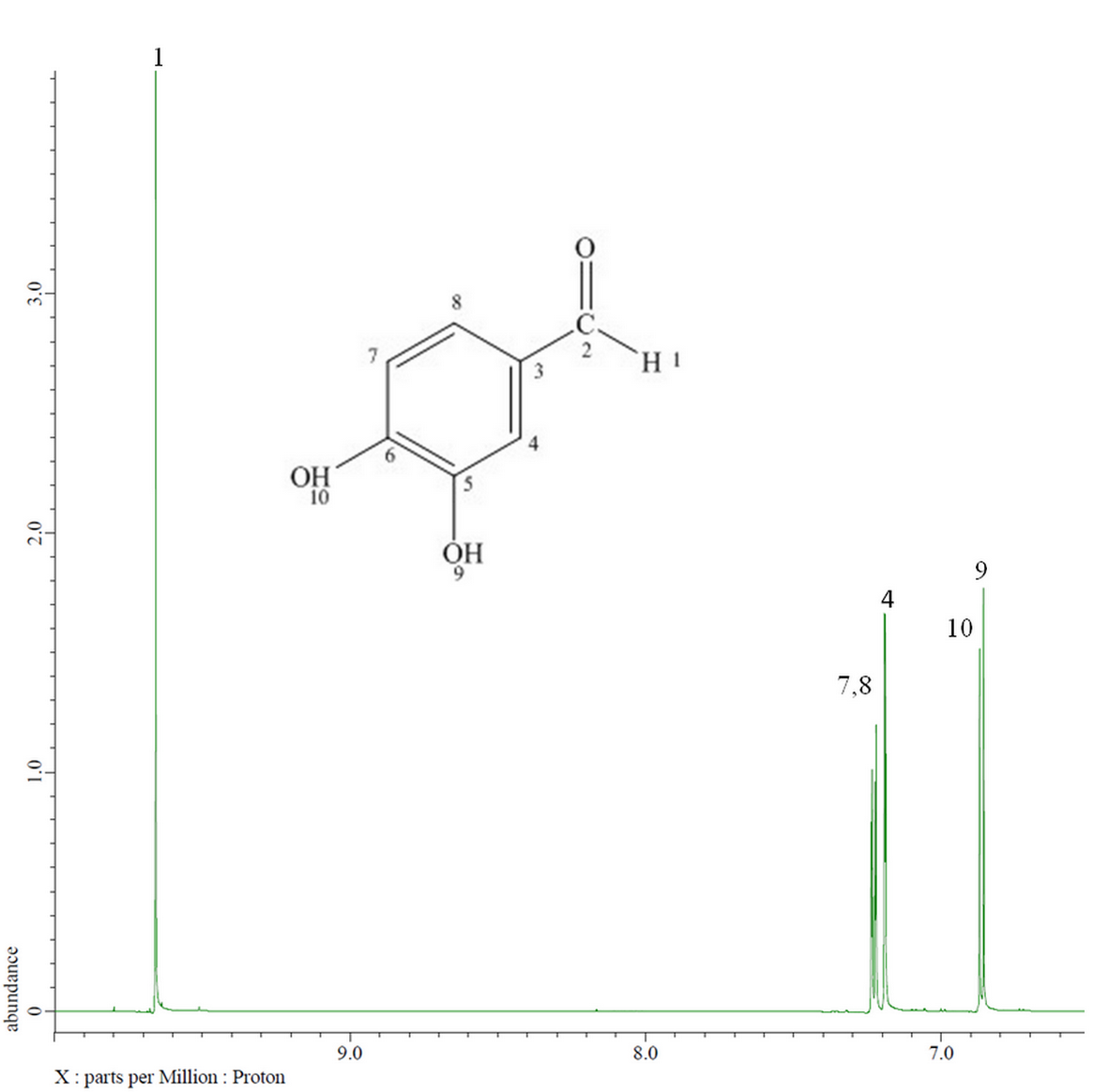

Supplement: S4 Fig — (TIF) [file pone.0122733.s004.tif]
